# Sinapine, but not sinapic acid, counteracts mitochondrial oxidative stress in cardiomyocytes

**DOI:** 10.1016/j.redox.2020.101554

**Published:** 2020-05-19

**Authors:** Doria Boulghobra, Pierre-Edouard Grillet, Mickaël Laguerre, Mathieu Tenon, Jérémy Fauconnier, Pascale Fança-Berthon, Cyril Reboul, Olivier Cazorla

**Affiliations:** a EA 4278, Laboratoire de Pharm-Ecologie Cardiovasculaire, Avignon University, Avignon, France; b PHYMEDEXP, INSERM, CNRS, Université de Montpellier, Montpellier, France; c Naturex SA, Science and Technology Department, Avignon, France

**Keywords:** Mitochondria, Natural antioxidant, Oxidative stress, Ischemia-reperfusion

## Abstract

**Introduction:**

When confronted to stress or pathological conditions, the mitochondria overproduce reactive species that participate in the cellular dysfunction. These organelles are however difficult to target with antioxidants. A feature of mitochondria that can be used for this is the negatively charged compartments they form. Most of mitochondrion-targeting antioxidants are therefore cationic synthetic molecules. Our hypothesis is that such mitochondriotropic traits might also exists in natural molecules.

**Aim:**

We tested here whether sinapine, a natural phenolic antioxidant-bearing a permanent positive charge, can target mitochondria to modulate mitochondrial oxidative stress.

**Methods:**

Experiments were performed *in-vitro*, *in-cellulo*, *ex-vivo,* and *in-vivo,* using cardiac tissue. The sinapic acid -lacking the positively-charged-choline-moiety present in sinapine-was used as a control. Sinapine entry into mitochondria was investigated *in-vivo* and in cardiomyocytes. We used fluorescent probes to detect cytosolic (H_2_DCFDA) and mitochondrial (DHR_123_) oxidative stress on cardiomyocytes induced with either hydrogen peroxide (H_2_O_2_) or antimycin A, respectively. Finally, ROS production was measured with DHE 10 min after ischemia-reperfusion (IR) on isolated heart, treated or not with sinapine, sinapic acid or with a known synthetic mitochondrion-targeted antioxidant (mitoTempo).

**Results:**

We detected the presence of sinapine within mitochondria *in-vitro,* after incubation of isolated cardiomyocytes, and *in-vivo,* after oral treatment. The presence of sinapic acid was not detected in the mitochondria. Both the sinapine and the sinapic acid limited cytosolic oxidative stress in response to H_2_O_2_. Only sinapine was able to blunt oxidative stress resulting from antimycin A-induced mtROS. Both mitoTempo and sinapine improved cardiac functional recovery following IR. This was associated with lower ROS production within the cardiac tissue.

**Conclusion:**

Sinapine, a natural cationic hydrophilic phenol, commonly and substantially found in rapeseed species, effectively (i) enters within the mitochondria, (ii) selectively decreases the level of mitochondrial oxidative stress and, (iii) efficiently limits ROS production during cardiac ischemia-reperfusion.

## Highlights

• Sinapine, a choline ester of sinapic acid, enters within mitochondria, whereas sinapic acid does not.
• Sinapine reduces mitochondrial oxidative stress, whereas sinapic acid does not.
• Sinapine reduces cardiac reactive oxygen species production during ischemia-reperfusion, whereas sinapic does not.

## Graphical abstract

**Figure:**
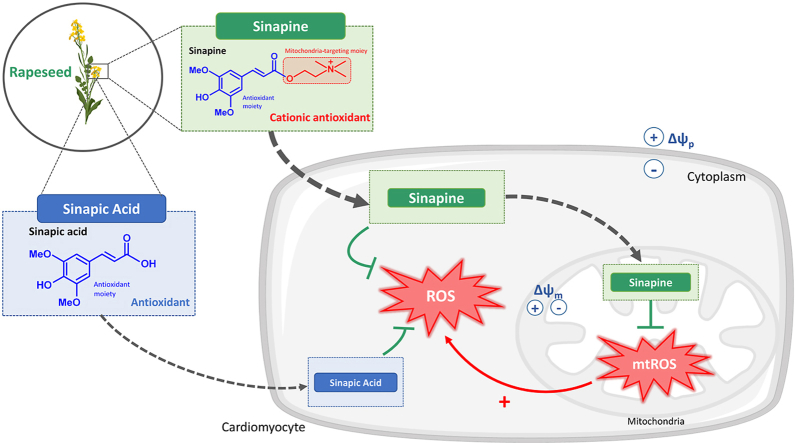

## 1 Introduction

Mitochondria are important intracellular organelles that are present in eukaryotic cells. Considering their key role in energy production and regulation of cell-stress-pathways, the mitochondria are critical targets when trying to understand tissue injury and disease. In the heart, the energy required to sustain the continuous contractile activity is almost exclusively provided by the mitochondria. In pathological conditions, the impaired mitochondrial electron transport chain (ETC) activity increases reactive oxygen species (ROS) production, leading to mitochondrial-driven injuries. Mitochondrial oxidative stress contributes to cell damages such as lipid peroxidation, proteins oxidation and DNA damages. The overproduction of mitochondrial ROS (mtROS) in the cardiovascular system results in proinflammatory cytokine production [1], hypertension [2], arrhythmia, contractile dysfunction, as well as hypertrophy, heart failure or ischemia-reperfusion injuries [3].

Accordingly, mitochondria constitute an important target for antioxidants to reduce mitochondrial oxidative stress, when endogenous defenses are overwhelmed. One limitation of this antioxidative strategy is to specifically target the mitochondria. Their specific property of forming negatively charged compartments can be used though. Actually, the electron transport chain activity creates a proton gradient across the inner mitochondrial membrane (ΔH^+^) that is used to drive the production of ATP by ATP synthase. The ΔH^**+**^ generates a mitochondrial membrane potential (ΔΨm) around -180 mV. Fifty years ago, the dibenzyl ammonium cation was shown to accumulate into the mitochondrial matrix according to this negative potential [4]. The mitochondrial tropism of some synthetic cations was then derived towards the specific development of antioxidants. Indeed, the cationic moiety—such as the triphenyl phosphonium cation (TPP^+^)—is covalently grafted to a neutral antioxidant, thus imparting the latter with mitochondrion-targeting properties. The high negative ΔΨm creates an electromotive force and thus offers a very selective way to deliver antioxidants to these organelles. Examples of such antioxidants targeting mitochondria include the TPP-ubiquinone, MitoQ [5], TPP-tocopherol [6], and MitoTEMPO [7]. These TPP-conjugates have been reported to significantly decrease the level of various mtROS. Considering the relative impermeability of biological membranes to hydrophilic molecules, hydrophobicity of compounds is also proposed as a key element to target mitochondria [8]. However, some hydrophilic chemical moieties, such as the choline ester -bearing a cationic charge covalently grafted to sulfhydryl compounds such as glutathione or N-acetyl-l-cysteine have been also proposed as mitochondrial targeting compounds [9]. These chemical modifications increase the ability of the resulting antioxidants to accumulate within the mitochondria and limit the oxidative damages [9,10].

Although many natural antioxidants have already been discovered, to our knowledge and so far, most mitochondrial selective antioxidants are synthetic compounds. There is however no reason to think that the chemical traits responsible for the mitochondrial targeting properties of synthetic compounds are not also present in some natural antioxidants. Herein, we report the identification of sinapine as a natural antioxidant with a mitochondrial tropism. This compound is present in substantial amounts in the *Brassicaceae* family, most especially in rapeseed and mustard. As a choline ester of the sinapic acid, the sinapine contains a quaternary amine that is permanently and positively charged that might confer to the sinapine a significant mitochondrial tropism. We therefore tested here the hypothesis that such a natural antioxidant, bearing a permanent positive charge, can enter within mitochondria to modulate mitochondrial oxidative stress when confronted to some stress situation. *In-vivo*, on isolated heart and on primary isolated rat cardiomyocytes, we found that the sinapine (i) enters within the mitochondria, (ii) decreases the levels mitochondrial oxidative stress, and (iii) prevents the overproduction of ROS when under stress.

## 2 Materials and methods

### 2.1 Animal studies

Male Wistar rats (12-week-old, n = 78; weight = 361 ± 4 g; Janvier, France) were housed with a 12-h light-dark cycle and free access to water and food. All investigations conformed to the European Parliament Directive 2010/63/EU and were approved by the local ethics committee (Comité d’éthique pour l'expérimentation animale Languedoc-Roussillon, n° CEEA-00322.03).

**Isolated cardiomyocytes** Ventricular cardiomyocytes were isolated as previously described [11]. Rats were euthanized using an i.p. injection of pentobarbital sodium (100 mg/kg) with heparin (100 U). The heart was rapidly excised, rinsed in an ice-cold Hanks–HEPES buffer (in mM NaCl 117, KCl 5.7, NaHCO_3_ 4.4, KH_2_PO_4_ 1.5, MgCl_2_ 1.7, HEPES 21, glucose 11.7, taurine 20, pH at 7.15), mounted on a Langendorff perfusion system and perfused (3 mL/min, at 37 °C), firstly with a Hanks–HEPES buffer for 5 min to be cleared from blood and then with a buffer supplemented with 1.2 mg/mL collagenase type 4 (Worthington, Lakewood, NJ, USA) for 13–18 min. Small pieces of left ventricle (LV) were dissected and mechanically dissociated. The Ca^2+^ concentration was gradually increased to 1 mM Ca^2+^ and cells were used within the day. Experiments were performed in Tyrode solution, with 1.8 mM Ca^2+^.

### 2.2 Fluorimetric detection of intracellular sinapine

The sinapine entry in the cardiomyocytes was measured using the sinapine autofluorescence. The cardiomyocytes were maintained in a laminin-coated Petri dish on a microscope stage (Axiovert, Zeiss, Germany; 20× objective). Cells were illuminated at 340 nm, using a lambda DG-4 excitation system (Sutter Instrument Company, CA, USA). Images were then captured digitally every 0.35 s with a cooled CCD camera (Photometrics, Roper scientific, France) programmed for 450 nm emission. Changes in fluorescence after correction for background were analyzed with the Metafluor software (Universal Imaging Corporation, USA).

### 2.3 HPLC quantification of cytosolic and mitochondrial levels of sinapine and sinapic acid

Antioxidants were also quantified in cardiomyocytes using a liquid chromatography-mass spectrometry (LC-MS/MS). Cardiomyocytes (100,000 cells/mL) were incubated in Tyrode for 1 or 2 h with different concentrations of sinapine (2, 20, and 60 μM) or sinapic acid (2, 20, and 60 μM). Extracellular, cytosolic and mitochondrial fractions were obtained and frozen in liquid nitrogen until HPLC measurement. After incubation, cells were pelleted by gravity and the supernatants collected for the extracellular fraction. The pellet of cells was rinsed twice in Tyrode. Cells were incubated with PBS containing digitonin (5 mg/mL) for 10 min on ice. Cells were then centrifuged at 10,000 g for 20 min at 4 °C, and the supernatants, consisting in the cytosolic fraction, was collected. The mitochondrial fraction was gathered after rinsing the pellets five times in PBS, to avoid the contamination of the cytosolic fraction.

These different fractions were then analyzed using an Agilent Poroshell EC-C18 120, 2.7 μm, 2.1 × 150 mm analytical column on an Agilent 1290 Infinity II series pump. Injection volume was 1 μL. HPLC flow rate was 0.3 mL/min with the following mobile phases: water with 0.1% formic acid (A) and acetonitrile with 0.1% formic acid (B). The gradient was as follows: 0.0–3.0 min 0–30% B, 3.0–7.0 min 30–60% B, then a stabilization step of 4 min at the initial condition. Autosampler tray temperature was set to 10 °C and column temperature was set to 25 °C. Detection was performed with an Agilent 6420 triple quadrupole in positive ionization mode with ESI source, gas temperature 325 °C, gas flow 9 L/min, nebulizer 35 psi, and capillary voltage 3500 V. Mass spectrometer was working in MRM mode acquisition, following the specific transition for isoproturon-d3 (internal standard 210.2->75.0), sinapine 310.2->251.1 (qualifier 175.0 and 91.0) and sinapic acid 225.1->207.0 (qualifier 91.0 and 65.0).

### 2.4 Antioxidant activity of sinapine and sinapic acid in cardiomyocytes

Cardiomyocytes (30,000 cells/mL) were incubated with sinapine or sinapic acid for 1 h. Thirty min before the end, a fluorogenic probe was added. We used dihydrorhodamine 123 (DHR_123_) to probe mitochondrial oxidative stress and 2′,7′-dichlorodihydrofluorescein diacetate (H_2_DCF-DA) to probe cytosolic oxidative stress. Both probes were used at 5 μM and were purchased at Invitrogen Molecular probes (Fischer Scientific). Cells were rinsed with a Tyrode solution and distributed into a microplate to test different conditions. Some cells were stimulated with a 0.1 mM H_2_O_2_ (Sigma Aldrich) or with a 10 μM of a complex III blocker, antimycin A (AA, Sigma Aldrich) to induce cellular mitochondrial ROS production. The microplate was immediately transferred to the microplate reader Tecan Infinite M200Pro. Fluorescence was measured at different wavelengths, every 5 min during 30 min.

### 2.5 Ischemia-reperfusion (IR) on isolated hearts

Ischemia-reperfusion on isolated hearts was performed as previously described [12]. The heart was perfused retrogradely using a Langendorff apparatus at a constant perfusion pressure (80 mmHg) and paced at 300 beats ⁄min (Low voltage stimulator, BSL MP35 SS58L, 3 V). A non-compliant balloon was inserted into the left ventricle to monitor the LV pressure. The hearts were then stabilized during 20 min, and perfused for 45 min -with or without sinapine (60 μM), sinapic acid (60 μM) or MitoTEMPO (Sigma Aldrich, 0.1 μM)-, then subjected to global no-flow ischemia for 20 min, followed by 10 min of reperfusion. During the entire IR procedure, cardiac functional parameters were recorded (MP36R, BioPac System Inc) and coronary effluents were collected to evaluate coronary blood flow levels.

### 2.6 Measurements of ROS production by DHE staining

After 10 min of post-ischemic reperfusion, LV samples were embedded in an Optimal Cutting Temperature (OCT from Tissue-Tek) and flash-frozen in liquid nitrogen. Frozen sections were covered with 10 μM dihydroethidium (DHE) and incubated in a light-protected humidified chamber at 37 °C for 5 min. Images were obtained with a fluorescence microscope (Olympus BX60, excitation: 488 nm; emission: 610 nm) at the 3 A INRA/University of Avignon imaging facility Department. A SOD-mimetic TEMPOL (10 mM, Santa Cruz Biotechnology) was used as a negative control to confirm that the signals obtained really resulted from the ROS production.

### 2.7 Sinapine bioavailability

To evaluate plasmatic and tissue-level bioavailability of sinapine, rats received 200 mg/kg of sinapine using oral gavage. Blood samples were collected 15, 30, 60, and 120 min after sinapine administration, from the right jugular vein. Sinapine and sinapic acid were then quantified in plasma using the following procedure: plasma (50 μL) is mixed with an internal standard solution (50 μL in methanol at 10 ng/mL) and vortexed during 1 min. The solution is then centrifuged during 3 min at 12,000 rpm, and the supernatant is injected into HPLC/MS for quantification.

At the end of the procedure, the animals were sacrificed, and hearts quickly removed. The right ventricle removed, and LV samples frozen in liquid nitrogen. The LV tissue was homogenized in a 2 mL mitochondria isolation buffer (in mM: 300 sucrose, 5 TES, 0.2 EGTA; pH 7.2), using a Polytron (Ultra Turrax T25, IKA Labortechnik). The homogenate was centrifugated at 1000 g during 10 min at 4 °C. To separate the cytosolic fraction from the mitochondrial one, the supernatant was centrifugated at 12,000 g during 15 min at 4 °C, then collected (cytosolic fraction), while the pellet was gathered for mitochondria fraction. To optimize the release of mitochondria materials, the pellet was homogenized in a 2% Triton-X100 lysis buffer during 10 min on ice. To remove Triton-X100, the mitochondrial fraction was washed, then centrifuged (12,000 g for 15 min at 4 °C) and the pellet was resuspended in a mitochondria isolation buffer. The cytosolic and mitochondria fractions obtained were quickly frozen in liquid nitrogen until HPLC measurement was performed. To do so, mitochondria was extracted using the same procedure as that used for plasma. The cytosol (50 μL) was pipetted into a 10 mL volumetric flask then spiked successively with an internal standard solution (200 μL at 250 ng/mL in methanol) and with 5 mL of methanol-water (50/50 v: v). The flask was ultrasonicated for 5 min and, once cooled down, completed up to 10 mL with methanol-water (50/50 v: v). Both the mitochondrial and the cytosolic fractions were analyzed with the LC-MS/MS procedure described above.

**Statistical analysis** Statistics were performed using the StatView 5.0 program (SAS Institute, Cary, NC). Data were expressed using the mean ± SEM. Differences were assessed with the one-way ANOVA, whenever appropriate. When significant interactions were found, a Bonferroni *post hoc* test was applied with p < 0.05. Fig. 2E; Fig. 4 and Fig. 5 statistics were obtained in a R-programming environment [13] (Available from: https://www.R-project.org/.) and using a linear-mixed-effect model *via* nlme [14].When significant interactions were found (p < 0.05), a false discovery rate *post hoc* test was applied.

## 3 Results

### 3.1 The subcellular distribution of the sinapine and sinapic acid in cardiomyocytes

**Fig. 1:**
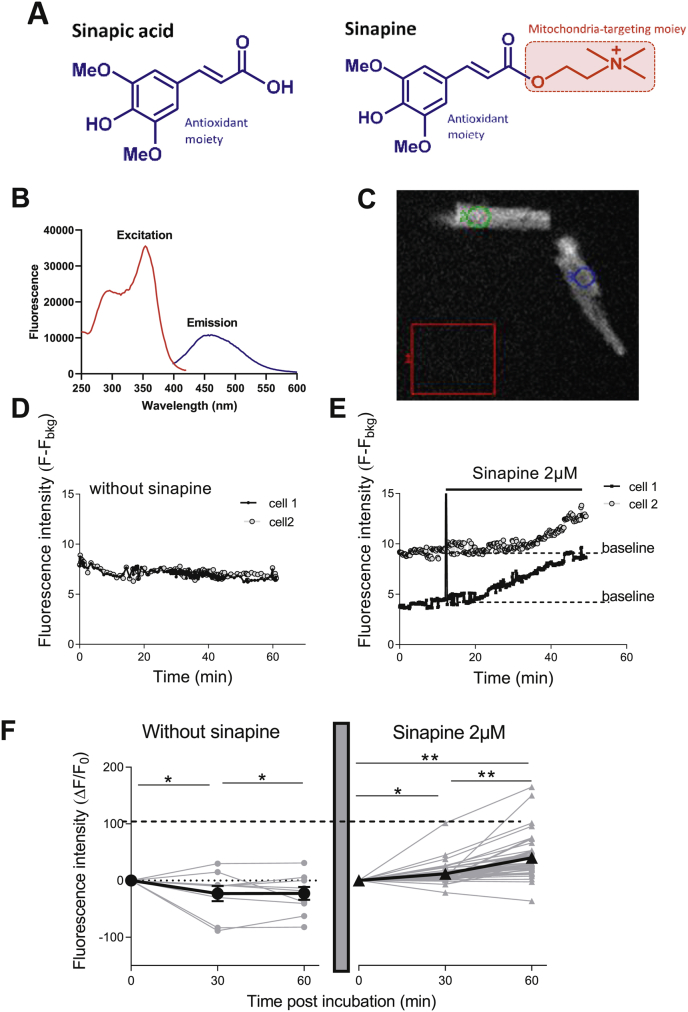
**Entry of sinapine in cardiomyocytes. (A)** Chemical structures of sinapic acid and sinapine identifying the potential antioxidant moiety in both compounds and the potential mitochondria-targeting moiety in sinapine. **(B)** Fluorescence spectrum of sinapine indicates a maximal excitation wavelength at 355 nm and a maximal emission wavelength at 455 nm. **(C)** Measurement of autofluorescence of sinapine using Metafluor System (Ex 340 nm/Em 450 nm) within cardiomyocytes after subtraction of the background in the red box 1 (F_bkg_). **(D**–**F)** Example of the stability of fluorescence (Ex 340 nm/Em 450 nm) of two cardiomyocytes in absence of sinapine. **(E)** When cardiomyocytes are incubated with 2 μM sinapine, the fluorescence within the cell increases suggesting sinapine intracellular accumulation. **(F)** Fluorescence of individual control cells after 30 min and 60 min (n = 10 control cells, left panel, grey circle) and after incubation with 2 μM sinapine (n = 41 cells, right panel, grey triangle). The average fluorescence of control (black circle) and sinapine (black triangle) incubated cardiomyocytes. Linear mixed model effect: p < 0.0001; Time: p < 0.0001; Group: p < 0.0001. *, p < 0.05, **, p < 0.01. (For interpretation of the references to colour in this figure legend, the reader is referred to the Web version of this article.)

Sinapic acid has been previously reported to be an orally bioavailable phytochemical [15]. As shown in Fig. 1A, sinapine is a choline ester of the sinapic acid containing a quaternary amine that is permanently and positively charged. Much less is known about the sinapine. Whether the sinapine constituted an antioxidant that can penetrate the cardiomyocytes and target the mitochondria was unknown.

**Fig. 2:**
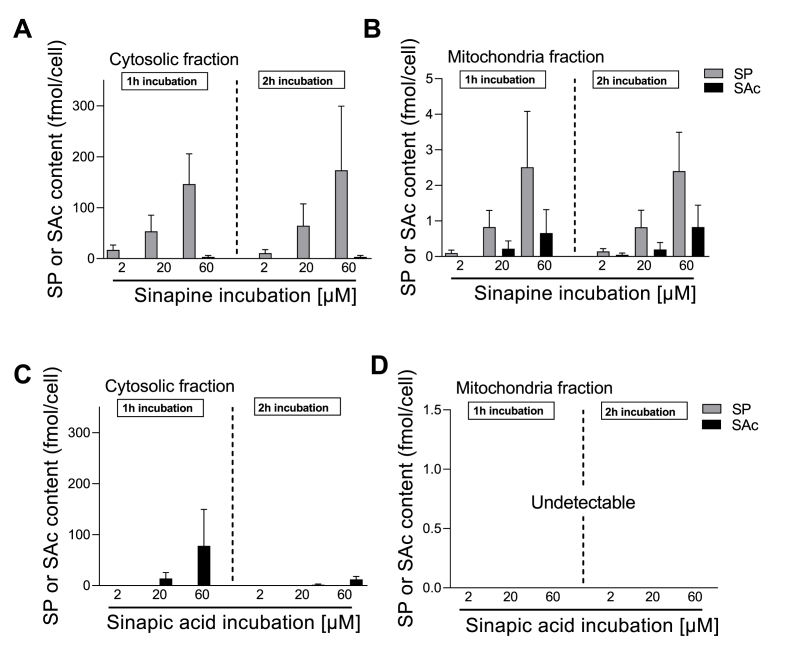
**Cytosol/mitochondria partitioning of sinapine and sinapic acid in cardiomyocytes.** Myocytes were incubated for 1 h (left panel) or 2 h (right panel) with different concentrations of sinapine **(A, B)** or sinapic acid **(C, D)** (2, 20, and 60 μM). After fractional separation, the amounts of sinapine (grey column) or sinapic acid (black column) were measured by HPLC in the cytosol **(A, C)** or in isolated mitochondria **(B, D).** Conditions without drug incubation served as a negative control. (n = 6 experiments with sinapine, n = 4 experiments with sinapic acid).

First, we evaluated whether sinapine was able to enter into freshly isolated adult cardiomyocytes. For this purpose, we used the autofluorescence properties of the sinapine (355 nm excitation/450 nm emission) (Fig. 1B). The fluorescence measured inside the cell (Fig. 1C) every min during 1 h decreased slightly with time, in control cells in absence of sinapine (Fig. 1D). Other cells were measured for 10 min to establish the baseline and then incubated with sinapine at a low dose (2 μM). Typically, fluorescence increased after 10–15 min, suggesting that sinapine was entering the cell (Fig. 1E–F). The fluorescence steadily increases over the 1 h-period tested (Fig. 1E–F).

To further evaluate the ability of antioxidants to accumulate into some specific myocyte compartments, in particular into the mitochondria themselves, we incubated intact cardiomyocytes with either sinapine (Fig. 2A and B) or sinapic acid (Fig. 2C and D) at three different concentrations (2, 20, 60 μM) during 1 and 2 h. The mitochondrial fraction was then separated from the cytosolic one, using a differential centrifugation. In each incubation condition and subcellular fraction, we measured the levels of both the sinapine and sinapic acid using LC-MS/MS. Indeed, it is expected that within the cells sinapine can be hydrolyzed into sinapic acid. On the contrary, normally the myocytes do not contain the sinapine esterase as in plants that can transform sinapic acid into sinapine.

After one or 2 h of incubation with sinapine, we detected into the cytosol the sinapine in a dose dependent manner, and almost no sinapic acid -indicative of minor sinapine hydrolysis (Fig. 2A). In Fig. 2B, sinapine was detected into the mitochondria, in a dose-dependent manner and similarly between 1 or 2 h of incubation. We also detected sinapic acid in this subfraction, suggesting that approximatively 25% of the sinapine taken up by the mitochondria was hydrolyzed after 1 or 2 h (Fig. 2B). The duration of the incubation had no impact on cellular sinapine levels. These compounds required less than 1 h to enter cardiac cells, which is a rather rapid transport.

In Fig. 2C and D, cardiomyocytes were incubated with only sinapic acid during 1 or 2 h. We also measured sinapine and sinapic acid in both cytosol and mitochondria. However, here sinapine content measurement was used as a negative control since normally the myocytes do not transform sinapic acid into sinapine. As expected, no sinapine was detected in those conditions. The sinapic acid was detected in the cytosol of the cardiomyocytes in a dose dependent manner, in particular after 1 h (Fig. 2C). The sinapic acid level measured in the cytosol (Fig. 2C) was lower than that of the sinapine (Fig. 2A). Interestingly, sinapic acid was not detected in the mitochondria, regardless of the incubation time and of the concentration. Altogether, the results indicate that, *in-vitro,* the sinapine can enter into the cardiomyocytes and localize both into the cytosol and the mitochondria, while the sinapic acid finds it more difficult to enter into the cardiomyocytes and only localizes into the cytosol.

**Fig. 3:**
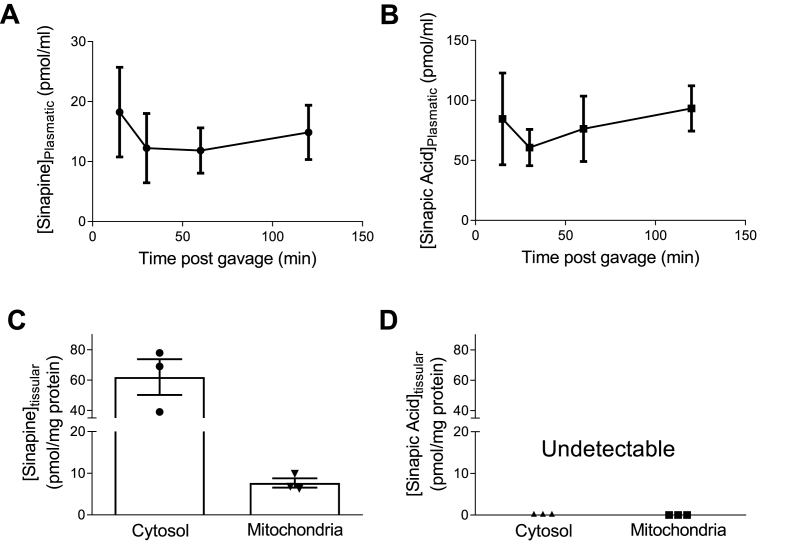
**Plasmatic and tissue levels of sinapine and sinapic acid after oral administration of sinapine (200 mg/kg) in rat.** Blood samples were collected at 10, 30, 60 and 120 min after oral administration of sinapine. Plasmatic levels of sinapine **(A)** and sinapic acid **(B)** were measured by LC-MS/MS. At the end of the 120 min, the heart was removed, rinsed and frozen. Differential centrifugation was performed to separate the mitochondrial fraction from the cytosolic fraction. The level of sinapine **(C)** and sinapic acid **(D)** were measured in each cellular subfraction by LC-MS/MS (n = 3 animals).

In order to verify that the sinapine could be found into both the plasma and the cardiac tissues after some oral administration at a human-compatible dose, we then performed *in-vivo* experiments in rats. Plasmatic and tissue levels of sinapine and sinapic acid were measured 10, 30, 60, and 120 min after oral sinapine administration (200 mg/kg) using LC-MS/MS (Fig. 3A–B). Sinapine and sinapic acid levels were detected into the plasma at all-times, pointing at a relatively stable level over the 2 h-period. We found about 5 times more sinapine-derived sinapic acid than sinapine in the plasma. About 120 min after gavage, the heart was removed, rinsed and quick-frozen into liquid nitrogen. Sinapine and sinapic acid contents were determined into the cytosolic and mitochondrial fractions as already mentioned (Fig. 3C–D). While sinapine was detected in both the cytosol and mitochondria (Fig. 3C), no sinapic acid, on the contrary, was found in any of the fractions (Fig. 3D). This showed that the orally administrated sinapine (i) is absorbed and consequently present into the blood stream, (ii) can reach the heart and, finally (iii) is able to enter both the cytosol and the mitochondria of cardiomyocytes.

**Fig. 4:**
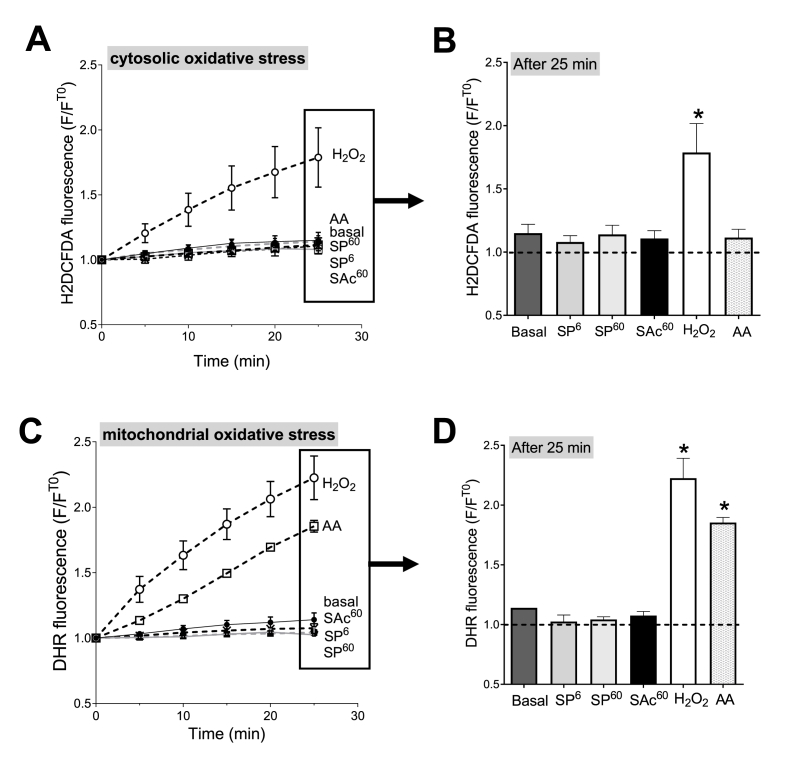
**H**_**2**_**O**_**2**_**and antimycin A-induced oxidative stress in cardiomyocytes.** Cardiomyocytes were loaded with fluorogenic dyes sensitive to ROS production. H_2_DCF-DA dye **(A, B)** probes oxidative stress within the cytosol. Dihydrorhodamine 123 (DHR_123_) dye **(C, D)** probes oxidative stress within the mitochondria. Fluorescence was measured every 5 min and was normalized by baseline fluorescence (T0) **(A, C)**. In control cells (basal), the fluorescence increases modestly during the 25 min-period. Some myocytes were incubated for 1 h with either 6 μM of sinapine (SP^6^), 60 μM of sinapine (SP^60^) or 60 μM of sinapic acid (SAc^60^) prior to measurement. Some cells were stimulated with 0.1 mM H_2_O_2_ or with a mitochondrial electron transport chain complex III blocker, antimycin A (AA, 10 μM) to force mitochondria to produce ROS. The fluorescence in each condition was compared with control cells (basal). **(B, D)** Representative average fluorescence after 25 min from graphs A and C. (n = 4 animals) (A, B) Linear mixed model effect: Time: p < 0.001, Group: p = 0.0139, Time-Group: p < 0.001. (C, D) Linear mixed model effect: Time: p < 0.001, Group: p < 0.001, Time-Group: p < 0.001. *, p < 0.05, **, p < 0.01.

**Fig. 5:**
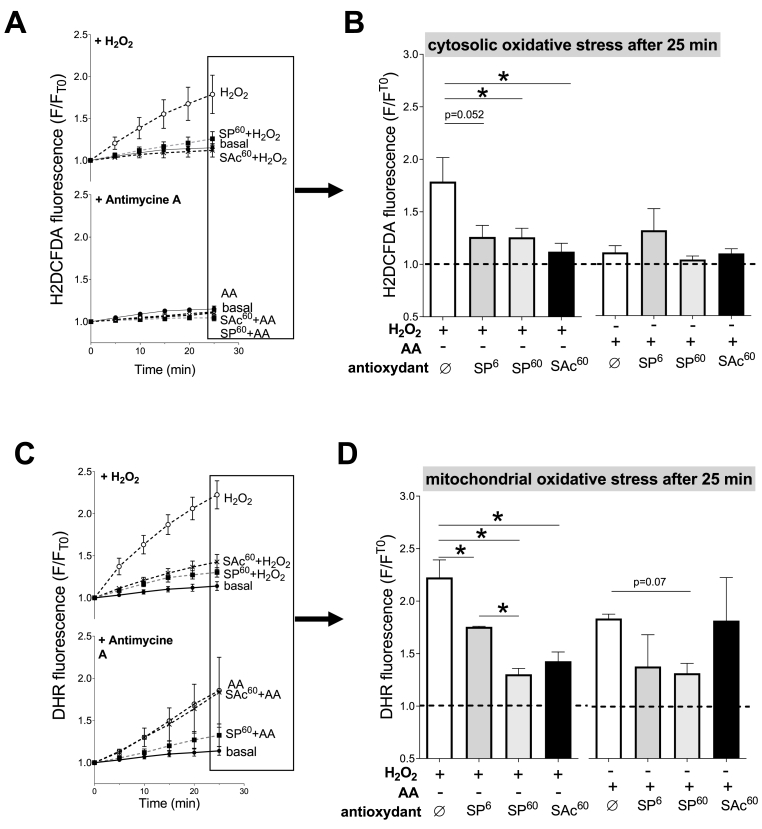
**Effect of sinapine and sinapic acid on the H**_**2**_**O**_**2**_**and antimycin A-induced oxidative stress in cardiomyocytes.** Cardiomyocytes were loaded with fluorescent dyes sensitive to ROS production. H_2_DCF-DA dye **(A, B)** probes oxidative stress within the cytosol. Dihydrorhodamine 123 (DHR_123_) dye **(C, D)** probes oxidative stress within the mitochondria. Fluorescence was measured every 5 min and was normalized by the baseline fluorescence (T0) **(A, C)**. Cardiomyocytes were stimulated with 0.1 mM H_2_O_2_ or with a mitochondrial electron transport chain complex III blocker, antimycin A (AA, 10 μM) to force mitochondria to produce ROS. Some myocytes were incubated for 1 h with either 6 μM of sinapine (SP^6^), 60 μM of sinapine (SP^60^) or 60 μM of sinapic acid (SAc^60^) prior to H_2_O_2_ and AA stimulation. The fluorescence in each condition was compared with control cells (basal). **(B, D)** Representative average fluorescence after 25 min from graphs A and C. *, p < 0.05 *vs* basal condition, (A, B) H2O2: Linear mixed model effect: Time: p < 0.001, Group: p = 0.0102, Time-Group: p < 0.001. Antimycin A: Time: p < 0.001, Group: p = 0.603, Time-Group: p = 0.528. (C, D) H2O2: Linear mixed model effect: Time: p < 0.001, Group: p = 0.0102, Time-Group: p < 0.001. Antimycin A: Time: p < 0.001, Group: p = 0.0016, Time-Group: p < 0.001. *, p < 0.05, **, p < 0.01.

### 3.2 The antioxidant activity of the sinapine against mitochondrial ROS production in isolated cardiomyocytes

Using two *in-vitro* assays, the ORAC (oxygen radical absorbance capacity) [16] and CAT (conjugated autoxidizable triene) [17] assays, we observed that both the sinapine (cationic) and the sinapic acid (lacking the cationic moiety) exert significant antioxidant activity (Suppl Fig. 1A and 1B, respectively). Considering the ability of the sinapine to accumulate within the mitochondrial subfraction, we evaluated its propensity to limit mitochondrial oxidative stress accumulation in isolated adult rat cardiomyocytes during a global or specific mitochondrial oxidative stress (Fig. 4). Two different ROS sensitive fluorogenic probes were used to detect specifically cytosolic (H_2_DCF-DA) or mitochondrial (DHR_123_) oxidative stress [18,19]. The myocytes loaded with one of the dyes were distributed into 96-well microplate. The fluorescence was determined every 5 min during 25 min (Fig. 4A and C). Some cells were incubated with either sinapine (6 or 60 μM) or sinapic acid (60 μM) 1-h prior measurement. After each incubation, the cells were washed out. Some cells were stimulated with 0.1 mM H_2_O_2_ for a global oxidative stress or with a mitochondrial electron transport chain complex III blocker -10 μM antimycin A (AA)- to force mitochondria into mtROS production [20]. Control cells (basal) *i.e.* without any exogenously added oxidant, showed that the fluorescence increase for both H_2_DCF-DA and DHR_123,_ during 25 min to be negligible (Fig. 4A and B). For control myocytes stressed with H_2_O_2_, H_2_DCF-DA (Fig. 4A and B) and DHR_123_ (Fig. 4C and D) the fluorescence continuously increased during 25 min, which indicates that cytosolic and mitochondrial oxidative stress occur in response to H_2_O_2_. In contrast, in AA-stressed control myocytes, H_2_DCF-DA fluorescence did not vary (Fig. 4A and B), while that of DHR_123_ increased continuously for 25 min (Fig. 4C and D), demonstrating that only mitochondrial oxidant species are produced when myocytes are incubated with AA. We can therefore confirm, in our experimental conditions, the ability of DHR_123_ to selectively detect mitochondrial oxidative stress and help discriminate a mitochondrial from a cytosolic one.

We then pre-incubated cardiomyocytes with some sinapine or sinapic acid before H_2_O_2_- or AA-induced stress was applied (Fig. 5). Pre-incubations with the two antioxidants alone had no impact on the fluorescence levels (Fig. 4B–D), suggesting no significant interactions between the sinapine or sinapic acid and the fluorescent probes used in the present work. When a cytosolic oxidative stress was generated by H_2_O_2_, both the sinapine (6 and 60 μM) and the sinapic acid (60 μM) prevented the H_2_DCF-DA (Fig. 5A & B) and DHR_123_ (Fig. 5C & D) fluorescence increase. The effect of the sinapine was a dose-dependent for the oxidative stress measured by DHR_123_. These results indicate that both the sinapine and the sinapic acid are efficient antioxidants when confronted to a global oxidative stress mediated by the cytosolic initiation of oxidation.

The AA conditions then allowed to test the antioxidant capacity of the sinapine and the sinapic acid under a specific mitochondrial oxidative stress. In presence of AA, the H_2_DCF-DA fluorescence was not affected in any condition, indicating no measurable cytosolic ROS production nor accumulation (Fig. 5A & B). On the contrary, the presence of AA induced an increase in the DHR_123_ fluorescence, which was efficiently prevented by only the sinapine. These last results strongly suggest that only sinapine is able to target mitochondrial oxidative stress.

The differential cardioprotective impact of the sinapine and sinapic acid in an isolated heart model for ischemia reperfusion.

**Fig. 6:**
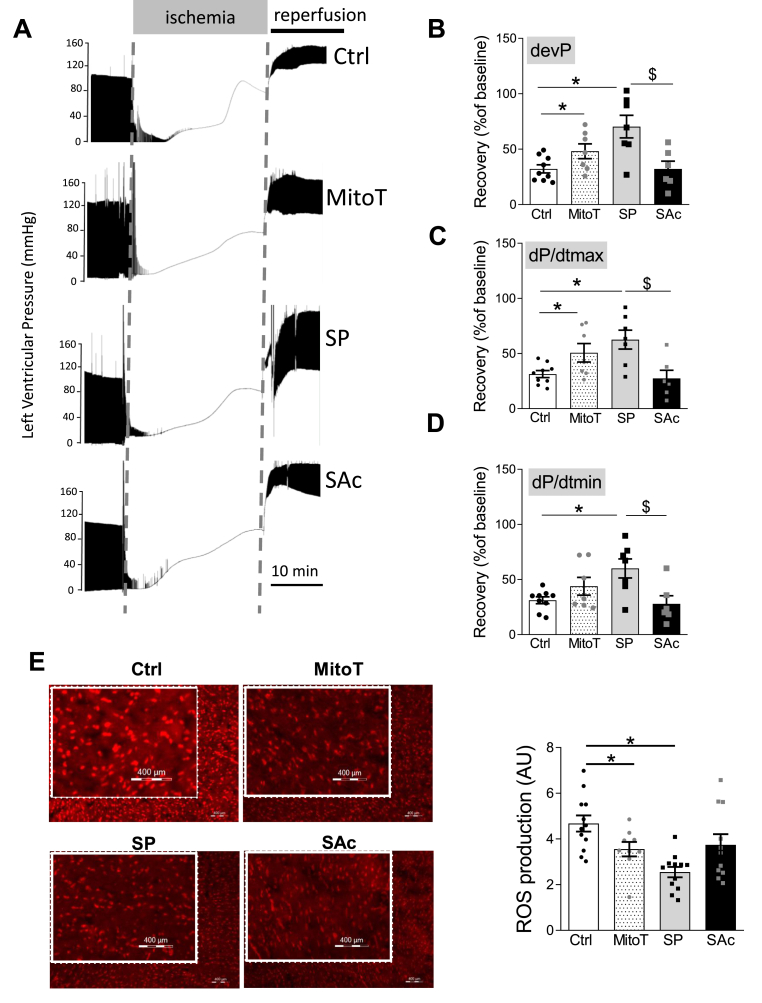
**Effect of sinapine and sinapic acid on cardiac functions following ischemia-reperfusion stress.** (A) Examples of LV pressure during the ischemia-reperfusion protocol of the isolated heart perfused retrogradely using a Langendorff apparatus. Hearts were stabilized for 20 min and then perfused for 45 min with or without sinapic acid (SAc 60 μM), sinapine (SP 60 μM) or MitoTEMPO (MitoT). Hearts were subjected to global no-flow ischemia for 20 min followed by 10 min of reperfusion. **(B**–**D)** Recovery of the developed pressure (Pdev), the maximal and minimal first derivative of left intraventricular pressure (dP/dtmax and dP/dtmin, respectively) 10 min post-IR. Values are normalized to the baseline level. **(E)** Determination of ROS production by DHE staining 10 min after IR, in hearts pre-treated or not with MitoTEMPO (0.1 μM), sinapine (60 μM), or sinapic Acid (SAc, 60 μM). Scale bar = 400 μm, (n = 12 control hearts, n = 9 MitoTEMPO hearts, n = 12 sinapine hearts, n = 11 sinapic acid heart). *p < 0.05.

We hereby evaluated whether the sinapine can protect the whole heart following an ischemic stress, using the standard global ischemia-reperfusion model in perfused isolated hearts (Fig. 6). In this model, mitochondrial oxidative stress is a key trigger of cardiac injuries and dysfunctions [21]. The potential cardioprotective effect of the sinapine (60 μM) was compared to that of the sinapic acid (60 μM) and to a known synthetic mtROS scavenger, the mitoTEMPO (MitoT, 0.1 μM) [22]. No major modification was observed during the ischemic phase with the different compounds. Typically, after 10 min reperfusion, the heart does not fully recover when compared with the baseline level. This is illustrated by an approximate 30% recovery only of the developed pressure (Pdev) (Fig. 6A and B), the maximal and minimal first derivative of the left intraventricular pressure (dP/dtmax and dP/dtmin, respectively) in control hearts. The Pdev (Fig. 6B) and dP/dtmax (Fig. 6C) recovery levels increased significantly up to 44–50% in presence of the mitoTEMPO, indicating a cardioprotection induced by the drug. The relaxation phase, dP/dtmin (Fig. 6D) was not affected by the mitoTEMPO. Interestingly, the cardioprotective effects induced by the sinapine were larger, with a parameters recovery reaching 60–70%. By contrast, the sinapic acid treatment had no effect on any recovery parameter when compared with control hearts. These functional results were related with ROS levels produced within the tissues after ischemia-reperfusion and measured by DHE staining on cardiac tissue (Fig. 6E). We observed that the mitoTEMPO reduced significantly DHE fluorescence when compared to control tissue, -by 24 %- indicating lower ROS accumulation during ischemia-reperfusion. Pre-treated sinapine hearts produced even less ROS, since DHE staining was reduced by 45%, when compared to control hearts. The sinapic acid had limited effects on ROS production and did not differ from the control tissues. Altogether, the results indicate that targeting mitochondrial oxidative stress can be used as a cardioprotective strategy that can be achieved using sinapine.

## 4 Discussion

In this study, we showed that the sinapine, a natural phenolic antioxidant bearing a permanent positive charge, can *in-vivo* and *in-vitro* penetrate cardiomyocytes. Despite its hydrophilicity, the sinapine can (i) localize within the cytosol and mitochondria, (ii) decrease the level of mitochondrial oxidative stress, (iii) improve cardiac functional recovery and limit ROS production during post-ischemic reperfusion.

Today, using the highly negative inner membrane potential of the mitochondria as an electromotive driving force to attract cationic molecules appears as an efficient strategy to target effectively the mitochondria with antioxidants, nitric oxide liberating molecules and probes (for review [23]). Such a negative membrane potential is not found in any other subcellular compartments and thus constitutes a very selective way to deliver compounds to these organelles. However, it is generally considered that the uptake of cations by the mitochondria is not only caused by the positive charge of the cation, but also by its hydrophobicity [24]. For example, the hydrophobicity of the tetraphenylphosphonium or that of the triphenylmethyl phosphonium is supposed to help them pass easily through the phospholipid bilayers of the mitochondria [8]. This pattern actually contrasts with that of the hydrophilic cations which cannot easily cross biological membranes, except when transport is facilitated by some ionophores or carrier proteins [24]. The relative impermeability of biological membranes to hydrophilic cations is supposedly largely due to the high demand in energy when moving an ion from an aqueous environment to the non-polar lipid interior of the membrane. Most mitochondriotropic antioxidants developed so far are therefore lipophilic cations. In our work we measured the partition of the sinapine, the sinapic acid, and the MitoTempo, a commercially available SOD mimetic with mitochondrial tropism, between a lipid phase and an aqueous phase, using a biphasic octan-1-ol/water mixture. As previously reported with other molecules bearing a choline ester group [9], sinapine is highly hydrophilic. Indeed, Log*D* (pH 7.0) used to evaluate its partition between the lipid and aqueous phases, is highly negative, meaning that the sinapine is almost totally distributed ( > 99.8%) in the aqueous phase. The sinapic acid can also be considered as hydrophilic, though to a lesser extent, while MitoTempo is amphiphilic and several MitoQ (MitoQ3, MitoQ5 and MitoQ10) whose Log*P* value is given in literature [25] are lipophilic (Suppl Fig. 1C). In our work, despite its strong hydrophilicity, the sinapine is able (i) to cross cell membranes and distribute into blood plasma after an oral administration, (ii) to be present in the cytosol of cardiac myocytes and (iii) finally, to be significantly taken up by the mitochondria. Interestingly, the sinapic acid, which is less hydrophilic than the sinapine (Log*D* (pH7.0) – 0.9 *vs.* – 3.0, respectively), crossed cellular membranes with less efficiency when incubated with cardiomyocytes than the sinapine at the same concentration levels (Fig. 2). Even more interesting is the observation made on the sinapic acid not being taken up by the mitochondria (Fig. 2D). These results based on subcellular fractionation were confirmed by the ability of the sinapine, but not of the sinapic acid, to prevent mitochondrial oxidative stress in presence of the AA (Fig. 5D). This also confirms that the choline moiety of the sinapine, together with its positive charge, is the main molecular determinant of the sinapine ability to enter into the mitochondria.

The energy barrier to enter mitochondria for hydrophilic compounds (whether or not they are cationic) is considered too high to allow for passive penetration. If the sinapine cannot enters passively into the mitochondria, it requires certainly a shuttle system. Several shuttle systems have been described to carry molecules into the mitochondria. The sinapine contains a positively charged choline moiety. Some hydrophilic cations such as metformin are taken up by specific mitochondrial cationic transporters to diffuse across the mitochondrial membranes [23]. Similarly, carnitine, that is a quaternary ammonium compound and is considered as chemical analogue of choline, is pushed into the mitochondrial matrix by a Carnitine Acyl-Carnitine Translocase [26]. Moreover, both cellular membranes and mitochondria contain choline transporters like-proteins (CTL1 and CTL2) responsible for the uptake of extracellular choline and organic cations [27]. CTL1 has been identified in heart tissue [28]. Thus, we speculate that thanks to structural homology, one of these carriers may facilitate the sinapine entry into the mitochondria and not the sinapic acid. Obviously, more experiments are required to determine exactly how the sinapine accumulates into the mitochondria. Alternatively, our results suggest that the hydrophilicity of a cation is not necessarily a limiting factor for its mitochondrial tropism. To the best of our knowledge, sinapine thus appears as the first reported natural hydrophilic free radical scavenger that can target cardiac mitochondria.

Another key result of this work is that sinapine is more efficient to improve cardiac functional recovery and reduce ROS production during post-ischemic reperfusion than sinapic acid, despite showing a slightly lower antioxidant activity (Suppl Fig. 1). It is now widely accepted that major damaging events during ischemic tissue reperfusion are due to some mtROS production burst during the first minute of post-ischemic reperfusion [29]. Indeed, mtROS can trigger oxidative damages and also, in a complex interplay with mitochondrial Ca^2+^ overload, promote the activation of the mitochondrial permeability transition pore (mPTP). This activation leads to the inhibition of the mitochondrial respiration and further cell death (for review [30]). In addition, mPTP activation also contributes to the mtROS production increase in a “ROS-induced ROS release” [31]. In the present study, the sinapine ability to specifically blunt mtROS accumulation can explain its higher efficiency over the sinapic acid. Other compounds used to target mtROS production, such as the mitoQ can reduce efficiently the heart sensitivity to IR [32]. In conditions mimicking the ischemia-reperfusion, the MitoTEMPO, a SOD mimetic TEMPOL moiety covalently conjugated to the lipophilic cation triphenylphosphonium, is also able to preserve mitochondrial integrity and attenuate necrosis and apoptosis to the same extent as SOD1 overexpression [33]. The MitoTEMPO can protect the cardiovascular system against hypertension, whereas the use of a similar dose of the non-targeted TEMPOL is ineffective [2]. This is in line with our differential results between the sinapine and sinapic acid, which indicate that targeting mitochondria is crucial for some pathologies. In our work, the perfusion of isolated hearts with the mitoTEMPO at concentration levels previously used to limit cardiac mtROS production [34], was also able to protect the heart during ischemia-reperfusion and to reduce global ROS production. This result, associated with the fact that the sinapine is prompter than the sinapic acid to protect the heart during IR, confirmed the key role of mitochondrial oxidative stress in IR injuries. Indeed, despite the fact that other sources of ROS may also contribute to IR injuries, like the xanthine oxidase, uncoupled nitric oxide synthase and NADPH oxidase [35,36] for example, the mtROS are considered as a precursor of cardiac IR injuries [37]. Accordingly, targeting specifically mitochondrial ROS production with mitoQ, which is established as a mitochondrial superoxide scavenger [38], has been reported to delay mPTP opening and improve cell survival in isolated cardiomyocytes subjected to hypoxia-reoxygenation [39] and also to provide an efficient strategy to limit IR injuries [32]. However, we cannot exclude in our work that the dual distribution of the sinapine in, both the cytosol and the mitochondria, could also contribute to its beneficial impact on cardio protection. The mitochondrial fraction of the sinapine molecules could inhibit the early phase of the mitochondrial oxidative stress, observed during the first minutes of the reperfusion, while the cytosolic fraction could limit the activation of other cellular oxidative pathways. Further studies will be needed to investigate this hypothesis.

The nature of the antioxidant mechanisms of the sinapine also remains to be explored. The fluorogenic probes used in our study detect changes in oxidative stress but are not informative for the exact nature of the radicals due to lack of ROS specificity. We can only rely on previous studies that explored the radical scavenging capacities of the sinapic acid toward superoxide or hydroxyradical [15]. Considering that both the sinapine and the sinapic acid share the same syringyl moiety responsible for the antioxidant action, we could speculate that the sinapine has also the ability to reduce those free radicals, which limit mitochondrial oxidative stress. Moreover, we cannot exclude that the beneficial effects of the sinapine are mediated by interactions with complexes of the mitochondrial ETC and decrease of ROS production. Indeed, some polyphenol extracts (flavonoids) have been found to inhibit ETC complexes in intact mitochondria [40]. However, another phenolic antioxidant, xanthohumol, induces generation of ROS and triggers apoptosis through inhibition of mitochondrial ETC complex I in human lung cancer cells [41]. Thus, indirect antioxidant effects of the sinapine resulting from its potential interaction with some mitochondrial ETC complexes cannot be excluded.

**Study limitation:** A limitation of our work is that we tested the use of the sinapine in a cardiac ischemic reperfusion injury model. However other pathological conditions associated with increased mitochondrial oxidative stress, include, but are not limited to cancer [42], heart failure [43], peripheral artery diseases [44], hyperglycemia [45], diabetes [46,47], insulin resistance, neurodegenerative diseases [48], chronic obstructive pulmonary diseases [49],hypertension [2] and inflammatory diseases [50] and could be explored. Finally, to further confirm the ability of sinapine to protect the heart, the therapeutic dose must be determined.

## 5 Conclusion

In summary, we hereby show that a phenolic antioxidant like the sinapine, bearing a permanent positive charge and partitioning almost totally (>99.8%) into the aqueous phase of a biphasic octan-1-ol/water mixture, can (i) enter into cells and mitochondria, (ii) selectively decrease the level of the mitochondrial reactive species in cardiomyocytes, and (iii) protect the heart against a severe ischemic stress involving mitochondrial defects. The fact that this particular antioxidant is obtainable from natural sources like the rapeseed or mustard, that it is bioavailable and distributes readily to the heart, raises the question of its potential usage in diets and of its nutritional effects. Our findings pave the way for a more systematic search on antioxidants targeting mitochondria in our diet, a strategy that has never been addressed before, to our knowledge. Yet, this could presumably help us identify a hitherto overlooked subfamily of food antioxidants (if any) with a considerable impact for future nutrition patterns. When these compounds are not provided by our diet, some dietary supplementations -based on sinapine or other phenolic choline esters-could then appear to be a promising nutritional alternative.

## Declaration of competing interest

The authors have declared no conflict of interest. Naturex is involved in the research of new ingredients for the food and nutraceutical industry.
