# Supplementary material for: Sinapine, but not sinapic acid, counteracts mitochondrial oxidative stress in cardiomyocytes
Source: Redox Biol. 2020 May 19;34:101554. doi: 10.1016/j.redox.2020.101554 (PMC7251366; doi:10.1016/j.redox.2020.101554)
Supplement: Multimedia component 1 [file mmc1.docx]

**Supplementary information**

**Sinapine, but not sinapic acid, counteracts mitochondrial oxidative stress in cardiomyocytes**

Doria Boulghobra^1^, Pierre-Edouard Grillet ^2^, Laguerre Mickaël^3^, Tenon Mathieu^3^, Jérémy Fauconnier ^2^, Pascale Fança-Berthon^3^, Cyril Reboul^1†^*, Olivier Cazorla^2, †^*

^1^, EA 4278, Laboratoire de Pharm-Ecologie Cardiovasculaire, Avignon University, Avignon, France.

^2^, PHYMEDEXP, INSERM, CNRS, Université de Montpellier, Montpellier, France

^3^, Naturex SA, Science and Technology Department, Avignon, France.

^†^, both senior co-authors and co-corresponding authors

* : **Corresponding Author:**

Olivier Cazorla, PhD

“Physiologie et Médecine Expérimentale du cœur et des muscles – PHYMEDEXP"

Inserm - CNRS – Université de Montpellier ; CHU Arnaud de Villeneuve, 34295 Montpellier cedex 05, France; Tel: (33)-4-67-41-52-44; Email: [olivier.cazorla@inserm.fr](mailto:olivier.cazorla@inserm.fr)

Cyril Reboul, PhD

Laboratoire de Pharm-écologie CardioVasculaire – EA4278; Faculty of Sciences, Avignon Université

74 rue Louis Pasteur, 84000 Avignon, France

Phone: +33 490162946 ; E-mail: [cyril.reboul@univ-avignon.fr](mailto:cyril.reboul@univ-avignon.fr)

Abbreviated title: Targeting mitochondria with a natural antioxidant

**Author Disclosure Statement:** No competing financial interest exists.

**Antioxidant activity measurement using the ORAC assay**

Experiments were conducted following the procedure of Ou et al. [1], wherein pure sinapine (Fortochem Technology Limited, Hong Kong) and sinapic acid (Sigma Aldrich) are solubilized in 50:50 (v/v) acetone : water mixture. Dilutions were prepared in a phosphate buffer saline (PBS, 75 mM, pH 7.0) for each tested molecule. Twenty-five microliters of these solutions and PBS alone (blank) were transferred into a 96-well microplate (BRAND, Germany). The plate was refrigerated, then introduced into the reader (Infinite, Tecan, Switzerland). A solution containing 150 µL of fluorescein (0.1 M) was added into each well. The plate was shaken for 8 s (2 mm amplitude) and incubated for 30 min at 37 °C. After zero value measurement, 25 µL of peroxy radical generator (2,2′-azobis (2-amidinopropane) dihydrochloride, AAPH solution (152 mM), were added into each well, and the decrease of fluorescein fluorescence (Excitation: 485 nm/emission: 535 nm) was monitored for 90 min every 90 s (60 measurements). The antioxidant value of a sample was calculated measuring the difference between the area under the curve of the sample and that of a blank without antioxidant. This gave the net area under the curve (AUC), which was then plotted on a graph as a concentration function. The slope of the linear part was then divided by the slope of the Trolox (standard) and calculated in the same conditions and analyzed on the same microplate. ORAC values were expressed as mol Trolox equivalent/mol of molecule.

**Antioxidant activity measurement using the CAT assay**

These experiments were conducted following the Laguerre et al. procedure [2], wherein sinapine and sinapic acid were solubilized in 100 μL DMSO, and then in 50:50 (v/v) acetone water mixture, at the desired concentration. Dilutions were then prepared in a phosphate buffer saline (PBS, 75 mM, pH 7.0) for each tested molecule. Fifty microliters of these solutions and PBS alone (blank) were transferred automatically into a 96-well microplate (Greiner, Austria). The plate was refrigerated (6 °C) before the sequence.

To prepare the tung oil in-water emulsion, twenty-five milliliters of a PBS solution -containing 34 μM Brij 35 (neutral emulsifier, estimated MW= 1198 g/mol)- were added to the 8 mg tung oil in a brown glass flask. This mixture was vortexed for 10 s, before its homogenization (Ultra Turrax), at approximately 2400 rpm for 90 s.

After introducing the microplate in the reader (Infinite, Tecan, Switzerland) and after 4 min of incubation at 37 °C, 100 μL of the tung oil-in-water emulsion (0.1 M) were added into each well. The plate was shaken for 60 s (2 mm amplitude). Then, 50 μL of the AAPH solution (4 mM) were added into each well, and the decrease of the tung oil absorbance was measured at 280 nm for 120 min, every 60 s (120 measurements).

The antioxidant value (CAT value) of a molecule was calculated using the same procedure as used for the ORAC assay and was expressed as mol Trolox equivalent/mol of molecule.

**Partition coefficient (Log*D* (pH 7.0)) measurement**

In a 10-mL glass tube, 2 mg of sinapine, sinapic acid or MitoTEMPO were added with a 4 mL 75 mM KH_2_PO_4_ buffer solution (aqueous phase) and 4 mL of octan-1-ol. The mixture was then vortexed for 1 hour, at room temperature, and left to equilibrate for 30 min for sinapic acid and sinapine, and overnight for MitoTEMPO. Both phases were collected with a Pasteur pipette and extemporaneously analyzed by LC-MS, using the procedure described below.

**Suppl Fig. 1. Physical chemical properties of sinapic acid and sinapine. (A-B)** Antioxidant activity of both compounds evaluated *in vitro* by **(A)** the oxygen radical absorbance capacity (ORAC) assay and **(B)** the conjugated autoxidizable triene (CAT) assay. **(C)** Logarithm of the measured partition coefficient (LogP) between octan-1-ol and water of sinapine, sinapic acid, and MitoTEMPO, and LogP values found in literature for MitoQ3, MitoQ5, and MitoQ10 [3].*p<0.05

**References**

[1] B. Ou, T. Chang, D. Huang, R.L. Prior, Determination of total antioxidant capacity by oxygen radical absorbance capacity (ORAC) using fluorescein as the fluorescence probe: First Action 2012.23, J. AOAC Int. 96 (2013) 1372–1376. <https://doi.org/10.5740/jaoacint.13-175>.

[2] M. Laguerre, L.J. López-Giraldo, J. Lecomte, B. Baréa, E. Cambon, P.F. Tchobo, N. Barouh, P. Villeneuve, Conjugated autoxidizable triene (CAT) assay: a novel spectrophotometric method for determination of antioxidant capacity using triacylglycerol as ultraviolet probe, Anal. Biochem. 380 (2008) 282–290. <https://doi.org/10.1016/j.ab.2008.06.006>.

[3] A.M. James, H.M. Cochemé, R.A.J. Smith, M.P. Murphy, Interactions of Mitochondria-targeted and Untargeted Ubiquinones with the Mitochondrial Respiratory Chain and Reactive Oxygen Species IMPLICATIONS FOR THE USE OF EXOGENOUS UBIQUINONES AS THERAPIES AND EXPERIMENTAL TOOLS, J. Biol. Chem. 280 (2005) 21295–21312. https://doi.org/10.1074/jbc.M501527200.
